# Distractor Inhibition Predicts Individual Differences in the Attentional Blink

**DOI:** 10.1371/journal.pone.0003330

**Published:** 2008-10-03

**Authors:** Paul E. Dux, René Marois

**Affiliations:** Department of Psychology, Vanderbilt Vision Research Center, Center for Integrative and Cognitive Neurosciences, Vanderbilt University, Nashville, United States of America; University of Sydney, Australia

## Abstract

**Background:**

The attentional blink (AB) refers to humans' impaired ability to detect the second of two targets (T2) in a rapid serial visual presentation (RSVP) stream of distractors if it appears within 200–600 ms of the first target (T1). Here we examined whether humans' ability to inhibit distractors in the RSVP stream is a key determinant of individual differences in T1 performance and AB magnitude.

**Methodology/Principal Findings:**

We presented subjects with RSVP streams (93.3 ms/item) of letters containing white distractors, a red T1 and a green T2. Subjects' ability to suppress distractors was assessed by determining the extent to which their second target performance was primed by a preceding distractor that shared the same identity as T2. Individual subjects' magnitude of T2 priming from this distractor was found to be negatively correlated with their T1 accuracy and positively related to their AB magnitude. In particular, subjects with attenuated ABs showed negative priming (i.e., worse T2 performance when the priming distractor appeared in the RSVP stream compared to when it was absent), whereas those with large ABs displayed positive priming (i.e., better T2 performance when the priming distractor appeared in the RSVP stream compared to when it was absent). Thus, a subject's ability to suppress distractors, as assessed by T2 priming magnitude, predicted both their T1 performance and AB magnitude.

**Conclusions/Significance:**

These results confirm that distractor suppression plays a key role in RSVP target selection and support the hypothesis that the AB results, at least in part, from a failure of distractor inhibition.

## Introduction

The ability to inhibit distractors is a key feature of successful goal-oriented behavior. Indeed, the failure to suppress irrelevant information can profoundly interfere with the processing of task-relevant stimuli[1]. Therefore, it is not surprising that distractor inhibition figures prominently in models of attention and information processing[2], [3].

Given its ubiquitous function in cognition, distractor suppression should be particularly important under conditions where distractors impair task performance. Such conditions occur in the attentional blink (AB) paradigm, which reveals humans' impairment in detecting or identifying the second of two targets (T2) in a rapid serial visual presentation (RSVP) stream of distractors if it appears within 200–600 ms of the first target (T1)[4]. The important contribution of distractors to the emergence of an AB is widely acknowledged[4]–8 and further underscored by the suggestion that individual variability in AB magnitude may be related to distractor processing[9]. However, despite previous work suggesting that inhibition is involved in RSVP target selection[10]–[12], the role of distractor suppression in the AB is not well established. Furthermore, the few studies that have examined this question have reached conflicting conclusions. While some authors propose that the suppression of distractors facilitates target selection and hence reduces the AB[13], others predict that sustained suppression, elicited by the post-T1 distractor, gives rise to the deficit[7].

The finding that people differ widely in AB susceptibility provides a powerful means to assess the role of distractor suppression in this attentional phenomenon[9]. To investigate the role of distractor inhibition, here we examined the relationships between T1 accuracy, the AB and the priming observed for T2 from a preceding distractor that shared the same identity as that of the second target (priming distractor). If subjects inhibit distractors, then the presence of the priming distractor should reduce T2 performance because, once suppressed (via the priming distractor) the T2 representation will be more difficult to reactivate when T2 is subsequently presented. By contrast, if distractors are not suppressed, the priming distractor should facilitate T2 report because the second target will benefit from its representation already being activated[14]. Thus, according to this distractor suppression hypothesis, subjects who exhibit modest ABs would be expected to demonstrate strong distractor suppression and hence reduced T2 priming, whereas subjects that manifest large ABs should exhibit weaker distractor suppression and hence more T2 priming. Finally, in addition to influencing T2 processing, distractor inhibition would also be expected to influence T1, as suppression should facilitate overall target selection. Thus, our key question concerns whether T2 distractor priming predicts individual differences in T1 accuracy and AB magnitude in a standard dual-target RSVP task.

## Materials and Methods

Forty-eight students (35 females) of Vanderbilt University participated. The Vanderbilt University Institutional Review Board approved the experimental protocol and informed written consent was obtained from the subjects after the nature and possible consequences of the study were explained to them. The experiment had a 2 (priming distractor present/absent)×2 (Lag4/10) repeated-measures design, and T1 and T2 given T1 correct (T2|T1) were the dependent variables.

**Figure 1:**
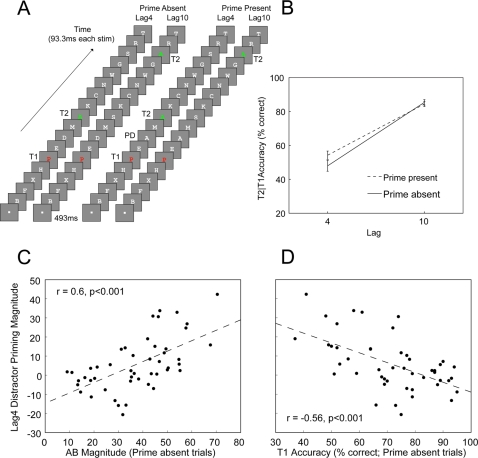
Experimental task and results. A) Subjects viewed RSVP streams of letters. Target 1 (T1) was coloured red, Target 2 (T2) green and the distractors white. T2 could appear at Lag4 or 10. In the prime present trials, a distractor (priming distractor, PD) with the same identity as T2 appeared at Lag2. All stimuli had different identities in the prime absent trials. Subjects were required to report T1 and T2 at the end of each RSVP stream. B) Effects of the priming distractor and Lag on T2|T1 accuracy. Errors bars represent standard error of the mean. C) Scatter plot of relationship between the AB (prime absent) and Lag4 distractor priming magnitude (T2|T1 % correct at Lag 4 in prime present trials – T2|T1 % correct at Lag 4 in prime absent trials). D) Scatter plot of relationship between Lag4 distractor priming magnitude and T1 accuracy (prime absent trials).

RSVP streams contained uppercase letters drawn from the alphabet excluding I, L, O, Q, U and V. T1 was red, T2 green, distractors white and the background grey. T1 appeared at serial position 5 and T2 at Lag4 or Lag10. A fixation square presented for 493 ms preceded all trials, while each stimulus appeared for 93.3 ms, with 17 of these stimuli presented in each trial. For the “prime absent” trials, all stimuli differed, while in the “prime present” trials the second distractor after T1 had the same identity as T2 (priming distractor; Figure 1A). This distractor appeared at the time of maximal blink (Lag2[5]) so that it was unlikely to be consciously perceived. Subjects typed the target identities when visually prompted at the conclusion of each stream. They performed 20 practice trials and 200 test trials, with the presentations of the four trial types randomly intermixed. The experiment was programmed in MATLAB using the Psychophysics toolbox[15], [16].

## Results and Discussion

A repeated-measures ANOVA with factors of Prime Presence/Absence and Lag demonstrated that T1 accuracy was not affected by any of the variables (*ps*>.26). By contrast, ABs were found in both the prime present and prime absent trials, with T2|T1 performance superior at Lag10 compared to Lag4, *F*(1,47) = 359.9, *p*<0.001. Replicating the priming results of Maki, Frigen and Paulson[17], in the group analysis the presence of the prime significantly enhanced T2|T1 accuracy at Lag4 but not Lag10, *F*(1,47) = 8.4, *p*<0.007 (Figure 1B). This finding that the AB was reduced when the priming distractor was present could be construed as evidence that distractors are not suppressed under RSVP conditions. However, a different picture emerges when considering individual differences in distractor priming and how they relate to T1 accuracy and AB magnitude.

To assess the contribution of distractor inhibition in accounting for individual differences in AB magnitude, and in RSVP target selection in general, we calculated the correlation between Lag4 distractor priming magnitude (T2|T1 prime present – T2|T1 prime absent) and both AB magnitude (Lag10 – Lag4 T2|T1) and T1 accuracy in prime absent trials. Distractor priming magnitude was assessed at Lag4 because of the short duration of RSVP priming[17]), while AB magnitude and T1 accuracy were only assessed in prime absent trials in order to get measures of T1 performance and the AB that were independent of the prime. Figure 1C shows that reduced priming (i.e., distractor suppression) was associated with attenuated ABs, *r*(46) = .6, *p*<0.001, suggesting that a failure of distractor suppression contributes to the deficit. In addition, a negative relationship existed between T1 performance and priming magnitude, *r*(46) = −.56, *p*<0.001 (Figure 1D), suggesting that inhibition also facilitates T1 selection. Importantly, distractor priming magnitude and the AB were still significantly correlated when T1 performance was partialed out, *r*(45) = .37, *p*<0.02, suggesting that the relationship between the AB and distractor priming does not simply reflect the influence of T1 processing on the AB. However, these results do not imply that subjects' ability to inhibit distractors is the only factor influencing AB performance: Indeed, T1 accuracy and the AB were negatively related even when distractor priming magnitude was controlled for, *r*(45) = −.45, *p*<0.002, as predicted by the hypothesis that subjects who are generally better at processing T1 exhibit smaller ABs[5].

**Figure 2:**
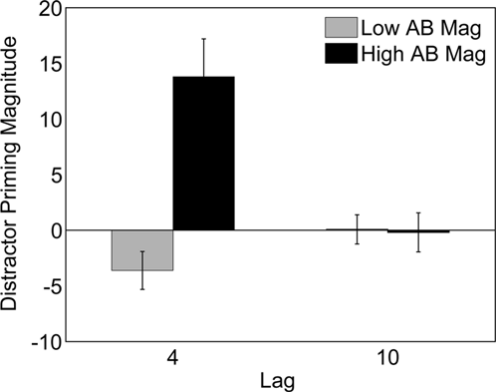
Priming magnitude in subjects with large and small ABs. Lag4 and Lag10 priming magnitude (T2|T1 % correct at Lag4/10 in prime present trials – T2|T1 % correct at Lag4/10 in prime absent trials) in the low (15 subs with the lowest AB magnitude: Low AB Mag) and high (15 subs with the highest AB magnitude: High AB Mag) AB magnitude groups. Errors bars represent standard error of the mean.

In a final analysis, we sought to confirm that subjects who exhibited reduced ABs did so because of distractor suppression rather than simply a failure to excite the distractor representations (i. e., they did not process the distractor stimuli). As previously discussed, if subjects actively inhibit distractors than the presence of the prime should impair T2 performance as this target's representation will be more difficult to reactivate once suppressed. Conversely, if subjects do not process distractors then there should be little to no effect of the prime on T2 performance, as the priming distractor will not activate this target's representation. To test this, we sorted our subjects based on the size of their AB in the prime absent condition, and compared the priming magnitude in those subjects with the smallest ABs (AB magnitude < = 30%, mean AB magnitude = 19%, n = 15) to those with the largest ABs (AB magnitude >46%, mean AB magnitude = 54.1%, n = 15). These two groups significantly differed in terms of AB magnitude, *t*(28) = 14.1, *p*<.001. Figure 2 shows that for subjects with large ABs the priming distractor led to superior T2|T1 performance at Lag4, *t*(14) = 5.1, *p*<.001, but not at Lag10 (*p* = .59). By contrast, for the subjects with reduced ABs the priming distractor significantly impaired T2|T1 accuracy at Lag4, *t*(14) = −2.1, *p* = .05, but not at Lag10 (*p* = .96). In addition, there was no effect of the prime at either Lag4 or Lag10 (*ps*>.25) in the remaining subjects with intermediate AB magnitude (mean AB magnitude = 38.6%, n = 18). Thus, it appears that subjects with reduced ABs, do indeed inhibit distractors. This is further support for the hypothesis that a failure of distractor inhibition contributes to the AB.

### Conclusion

Individual differences in T1 accuracy and AB magnitude can, in large part, be accounted for by distractor suppression: Subjects with high T1 accuracy and attenuated ABs inhibit distractors, whereas subjects with low T1 accuracy and large ABs do not (or do so to a lesser extent). These findings underscore the importance of examining individual differences in task performance to understand cognitive processes[9], as the role of distractor suppression in the AB was obscured in the group data (see Fig 1B). These results also confirm a key role for distractor suppression in RSVP target selection[10]–[12] and support the hypothesis that the AB results, at least in part, from a failure of inhibition[13] rather than sustained suppression elicited by the post-T1 distractor[7], for increased distractor inhibition was associated with attenuated ABs. More generally, our findings fold the AB in a wide class of attentional phenomena that depend on inhibition[18], thereby extending the role of distractor suppression to the temporal control of attention.
